# Perception of Mental Health Care Professionals in Saudi Arabia on Computerized Cognitive Behavioral Therapy: Observational Cross-sectional Study

**DOI:** 10.2196/26294

**Published:** 2021-05-03

**Authors:** Ahmad N AlHadi, Khawla A Alammari, Lojain J Alsiwat, Nojood E Alhaidri, Nouf H Alabdulkarim, Nouf A Altwaijri, Shamma A AlSohaili

**Affiliations:** 1 Department of Psychiatry College of Medicine King Saud University Riyadh Saudi Arabia; 2 SABIC Psychological Health Research & Applications Chair Department of Psychiatry, College of Medicine King Saud University Riyadh Saudi Arabia; 3 College of Medicine King Saud University Riyadh Saudi Arabia

**Keywords:** CBT, iCBT, cCBT, knowledge, attitude, mental health care professionals, computer usage, psychotherapy, therapy, cognitive behavioral therapy, health care worker, perception, Saudi Arabia, preference, mental health

## Abstract

**Background:**

Mental health disorders are common in Saudi Arabia with a 34% lifetime prevalence. Cognitive behavioral therapy (CBT), a type of psychotherapy, is an evidence-based intervention for the majority of mental disorders. Although the demand for CBT is increasing, unfortunately, there are few therapists available to meet this demand and the therapy is expensive. Computerized cognitive behavioral therapy (cCBT) is a new modality that can help fill this gap.

**Objective:**

We aimed to measure the knowledge of cCBT among mental health care professionals in Saudi Arabia, and to evaluate their attitudes and preferences toward cCBT.

**Methods:**

This quantitative observational cross-sectional study used a convenience sample, selecting mental health care professionals working in the tertiary hospitals of Saudi Arabia. The participants received a self-administered electronic questionnaire through data collectors measuring their demographics, knowledge, and attitudes about cCBT, and their beliefs about the efficacy of using computers in therapy.

**Results:**

Among the 121 participating mental health care professionals, the mean age was 36.55 years and 60.3% were women. Most of the participants expressed uncertainty and demonstrated a lack of knowledge regarding cCBT. However, the majority of participants indicated a positive attitude toward using computers in therapy. Participants agreed with the principles of cCBT, believed in its efficacy, and were generally confident in using computers. Among the notable results, participants having a clinical license and with cCBT experience had more knowledge of cCBT. The overall attitude toward cCBT was not affected by demographic or work-related factors.

**Conclusions:**

Mental health care professionals in Saudi Arabia need more education and training regarding cCBT; however, their attitude toward its use and their comfort in using computers in general show great promise. Further research is needed to assess the acceptance of cCBT by patients in Saudi Arabia, in addition to clinical trials measuring its effectiveness in the Saudi population.

## Introduction

Mental health disorders are very common in Saudi Arabia, which are present in approximately 34% of the general population [1]. Cognitive behavioral therapy (CBT) is a talk-based psychotherapy that can aid in managing psychological problems by changing thinking and behavior. Research has shown that the demand for CBT is increasing [2]. Unfortunately, there are few therapists available to meet the demand and high cost [3]. To fill the gap caused by the increasing demand and low supply for CBT services, the spectrum of CBT self-help resources is growing, ranging from self-help books [4] to self-help guided programs delivered via technology-based interventions, which have been used in health care settings.

Computerized cognitive behavioral therapy (cCBT) provides a flexible health care delivery process in which patients can start their therapy with a low-intensity intervention involving only limited practitioner support. cCBT provides many advantages for the user, such as flexibility and privacy, as patients can start cCBT at any favorable setting and time [5]. Furthermore, cCBT can be delivered with or without therapist guidance [6]. In the United Kingdom, the application of conventional one-on-one CBT is constrained by the limited number of qualified therapists, with wait times for one-on-one CBT ranging between 6 months and 2 years [7]. This limitation has driven the development of self-applied CBT alternatives such as self-help books and electronic-based programs [7].

Technology-based interventions and computer-aided psychotherapy, including virtual apps and internet-based solutions, provide an attractive alternative in digitally developed countries such as Saudi Arabia, where most of the population has access to computers and mobile phones [7]. A cross-sectional study performed by the Communications and Information Technology Commission in Saudi Arabia showed that among 3000 participants aged between 12 and 65 years, 73% had access to a desktop, laptop, or tablet computer [8]. In particular, cCBT provides substantial accessibility, scalability, and flexibility benefits over conventional one-on-one CBT, and has been suggested as an effective treatment [7,9-16], especially for low-intensity interventions [7,17]. Several previous studies have displayed great diversity in the types of mental health care professionals included for evaluation, with some covering psychologists [18] or young professionals [19] exclusively, and subsequent studies covering an extended spectrum of mental health care professionals [20]. Most mental health care professionals appear to consider cCBT as inferior to one-on-one therapy, with 17%-33% declaring that cCBT can yield similar results to traditional practice [18,20,21].

Moreover, most mental health care professionals consider that therapy using computers is better utilized for protection and for mild to moderate psychological conditions [20-23]. However, a notable percentage of mental health care professionals consider the use of cCBT in their future management plans, showing a positive attitude about the promising involvement of cCBT [18,20,21]. The knowledge of cCBT among mental health professionals is generally low [7,24], implying that knowledge of the effectiveness and availability of programs requires further development [18,19,23]. Detailed investigations revealed that some mental health care professionals were ill-informed regarding cCBT programs and the research behind them [25]. Clinician attitudes toward cCBT and other computer-based therapies were investigated in different countries such as Australia [22,23] and the United States [18,24,26,27]. Regardless of the varying results from study to study, some similarities have been found [25].

There is a rising interest in mental health care professionals’ attitudes toward cCBT as a vital additional treatment modality [25]. However, no studies have been performed to date on cCBT in Saudi Arabia. In this study, we aimed to measure the knowledge of cCBT, evaluate the attitude and preference toward cCBT, and determine the usage of computers among mental health care professionals in Saudi Arabia, including psychiatrists, psychologists, and others.

## Methods

### Setup, Sampling, and Process

A quantitative observational cross-sectional study was performed. We translated the knowledge assessment test from Donovan et al [25]. For attitude assessment, we used the translated version the questionnaire applied in the study of Becker and Jensen-Doss [28] after obtaining approval from the authors. The translation process followed the guidelines detailed in Sousa and Rojjanasrirat [29].

This study targeted health care workers who could potentially deliver cCBT such as psychiatrists, psychologists, and social workers, among others, and was carried out between February and March of 2018 on a clustered sample spanning tertiary hospitals in Riyadh, Saudi Arabia. Due to poor responses and limited resources, an expanded data collection to span electronically collected data from mental health professionals in the Kingdom of Saudi Arabia was needed; therefore, we altered the sampling technique to convenience sampling. Similar to the Australian study results [25], the target sample size was calculated using the following equation by setting *Z_α_* to 1.282, *S* to 1.48, and *d* to 0.2, to obtain 90% confidence: n=*Z_α_*^2^ *S*^2^/*d*^2^=(1.282(^2^) 1.48(^2^/(0.2)^2^=90, where *Z_α_* is the normal deviate, reflecting the type I error (calculated for 80% error); *S* is the standard deviation; and *d* is the accuracy of the estimate.

Considering a nonresponse rate of 30%, the total target sample size was 117 participants (90+27). With relaxation of the sampling technique, a total sample size of 121 participants was reached. A pilot study was performed with 10 mental health care professionals at King Khalid University Hospital in Riyadh (who were not included in the sample) to estimate the time needed to fill out the survey, assess the questionnaire’s comprehensibility, and determine any additional logistical requirements.

### Ethical Considerations

All participants were informed of the purpose of the study and their right to withdraw at any time without any obligation toward the study team via a consent form. No incentives or rewards were provided for participation. The study design and purpose of the study were approved by the King Saud University Institutional Review Board before data collection commenced.

### Questionnaire

#### Design

The questionnaire was designed as a self-administrated electronic form to maximize the response rate and cover multiple demographics.

#### Knowledge Scale

Knowledge was measured using six statements regarding computerized interventions. Participants chose between options of “true,” “false,” or “unsure.” This scale was adapted from Donovan et al [25].

#### Attitude Scale

Attitude and comfort toward computer-assisted therapy were measured using the Computer-Assisted Therapy Attitudes Scale [28]. This is an 11-item questionnaire that measures efficacy (belief in efficacy) and comfort using a 5-point Likert scale ranging from 1 (strongly disagree) to 5 (strongly agree). We reversed the scores for negative items; thus, in all cases, higher scores indicate a more positive attitude.

#### Demographics and Work-Related Questions

The third part of the questionnaire included demographics and work-related questions such as those related to CBT experience and clinical license.

### Data Analysis

Data were analyzed using Statistical Package for Social Sciences version 22 (IBM Corp). Continuous variables such as age are expressed as mean (SD), whereas categorical variables are expressed as frequency and percentages. The *t* test and one-way analysis of variance were used to compare continuous variables. A *P* value <.05 was considered statistically significant.

## Results

The questionnaire was sent to 132 mental health care professionals. We excluded 10 participants from the final analysis because they were also involved in our pilot study. Only one person refused to participate in the survey, and we had a near 100% completion rate from the participants who started the electronic survey, for a total of 121 participants, most of whom were women (73/121, 60.3%). The demographic characteristics of the participants, and the mean total scores for knowledge about cCBT and the feeling of using computers in therapy are summarized in Table 1. There was no statistically significant difference in knowledge with respect to demographic and work-related factors except for having a license and experience with cCBT. Knowledge was higher for clinicians who are licensed and who had experience with cCBT.

To measure the knowledge of cCBT, a test of cCBT facts was used, and the results are summarized in Table 2. The test is composed of six statements about computerized interventions, and the participants could answer with “true,” “false,” or “unsure.” The sum of answered items reflects the knowledge of cCBT; the higher the scores, the greater the knowledge of cCBT. The knowledge scale showed fair internal reliability, with Cronbach α=.601. Most of the participants showed uncertainty and lack of knowledge.

The results assessing the mental health care professionals’ feelings toward using computer-assisted therapy programs are summarized in Table 3. The attitude scale showed good internal reliability with Cronbach α=.819.

**Table 1:** Demographic characteristics of the participants, and total scores for knowledge about computerized cognitive therapy and feelings toward using computers in therapy (N=121).

Characteristic			Value		Knowledge about computerized cognitive therapy				Feeling comfortable toward using a computer in therapy		
					Correlation coefficient or mean (SD)^a^		*P* value		Correlation coefficient or mean (SD)^b^		*P* value
Age (years), mean (SD), median			36.55 (9.11), 37.00		–0.040		.67		0.051		.58
**Gender, n (%)**							.31				.16
	Female	73 (60.3)		0.86 (1.28)				37.07 (6.16)			
	Male	48 (39.7)		0.79 (1.03)				37.71 (4.92)			
**Specialty, n (%)**							.69				.32
	Psychiatry	29 (23.97)		1.04 (1.21)				37.43 (4.24)			
	Psychology	80 (66.12)		0.76 (1.19)				37.01 (6.00)			
	Sociology	8 (6.61)		1.00 (1.20)				41.25 (6.96)			
	Nursing	2 (1.65)		1.00 (1.41)				35.00 (4.24)			
	Other	2 (1.65)		0.00 (0.00)				35.00 (5.66)			
**Area of residence, n (%)**							.72				.13
	Riyadh	80 (66.67)		0.80 (1.16)				36.51 (5.70)			
	Makkah	19 (15.83)		0.74 (1.10)				39.58 (5.59)			
	Madinah	3 (2.50)		0.33 (.58)				35.33 (7.23)			
	Eastern	12 (10.00)		1.33 (1.61)				39.25 (4.65)			
	Aser	2 (1.65)		1.50 (2.12)				35.00 (0.00)			
	Jazan	2 (1.67)		0.50 (0.71)				43.00 (4.24)			
	Al Baha	2 (1.67)		0.50 (0.71)				41.50 (0.71)			
**Primary work setting, n (%)**							.23				.48
	Public Hospital	49 (40.50)		0.980 (1.41)				36.47 (5.80)			
	Psychiatric hospital	24 (19.83)		1.08 (1.28)				38.76 (5.01)			
	Primary care center	5 (4.13)		1.00 (0.70)				39.60 (4.28)			
	Home care facilities	13 (10.74)		0.31 (0.48)				37.23 (7.79)			
	Other	30 (24.79)		0.60 (0.89)				37.20 (5.16)			
**Professional license, n (%)**							.01				.07
	Yes	112 (92.6)		0.88 (1.21)				37.22 (5.49)			
	No	9 (7.44)		0.22 (0.44)				38.67 (8.00)			
**Education level, n (%)**							.12				.75
	Fellowship/board	20 (16.53)		1.20 (1.32)				37.10 (4.53)			
	PhD	6 (4.96)		0.33 (0.52)				39.83 (4.54)			
	Master	40 (33.06)		1.03 (1.49)				37.20 (7.30)			
	Bachelor	55 (45.45)		0.62 (0.85)				37.23 (4.85)			
**Previous use of a computer-assisted therapy program, n (%)**							.001				.32
	Yes	7 (5.79)		2.57 (1.13)				39.43 (5.16)			
	No	114 (94.21)		0.73 (1.11)				37.20 (5.71)			

^a^Based on a total possible score of 6.

^b^Based on a total possible score of 55.

**Table 2:** Knowledge about computerized cognitive behavioral therapy (N=121).

Question (correct answer)	True, n (%)	Unsure, n (%)	False, n (%)
Computerized interventions are only available online (False)	9 (7.4)	83 (68.6)	29 (24.0)
All computerized interventions involve therapist contact (False)	32 (26.4)	70 (57.9)	19 (15.7)
Computerized interventions are less effective than face-to-face therapy (False)	36 (29.8)	76 (62.8)	9 (7.4)
Computerized interventions automatically tailor to individual needs (False)	31 (25.6)	81 (66.9)	9 (7.4)
People who receive computerized interventions are generally satisfied (True)	12 (9.9)	106 (87.6)	3 (2.5)
Computerized interventions are not interactive (False)	20 (16.5)	78 (65.4)	23 (19.0)

**Table 3:** Therapist attitudes and access to computer-assisted therapy (N=121).

Question	Strongly disagree, n (%)	Disagree, n (%)	Neither agree nor disagree, n (%)	Agree, n (%)	Strongly agree, n (%)
If given the opportunity and training, I would like to use computers in therapy	1 (0.8)	2 (1.6)	17 (13.9)	66 (54.1)	36 (29.5)
I feel apprehensive about using computers during therapy	28 (23.0)	42 (34.4)	39 (32.0)	11 (9.0)	2 (1.6)
I am afraid that if I begin to use computers, I will become dependent upon them and lose some of my own skills	18 (14.8)	48 (39.3)	34 (27.9)	18 (14.8)	4 (3.3)
Using computers in therapy will interfere with rapport	5 (4.1)	30 (24.6)	48 (39.3)	32 (26.2)	7 (5.7)
My clients will be more likely to drop out of treatment if I use a computer program as part of the therapy	9 (7.4)	42 (34.4)	49 (40.2)	22 (18.0)	0 (0)
My clients would find it engaging to learn new skills using a computer	0 (0)	6 (4.9)	50 (41.0)	57 (46.7)	9 (7.4)
I believe that using computer programs in therapy will lead to better outcomes for my clients	0 (0)	9 (7.4)	56 (45.9)	47 (38.5)	10 (8.2)
The challenge of learning about the use of computers in therapy seems overwhelming to me	9 (7.4)	47 (38.5)	46 (37.7)	20 (16.4)	0 (0)
I am confident that I can learn the skills to use computer-assisted therapy	1 (0.8)	2 (1.6)	14 (11.5)	80 (65.6)	25 (20.5)
My clients are not sufficiently computer savvy to use computers in therapy	0 (0)	21 (17.2)	52 (42.6)	46 (37.7)	3 (2.5)
I have sufficient access to computers to use them in sessions	19 (15.6)	42 (34.4)	33 (27.0)	22 (18.0)	6 (4.9)

## Discussion

### Principal Findings

Our study assessed mental health care professionals’ knowledge and attitudes toward cCBT. Most of the participants were uncertain and lacked knowledge. However, the majority of participants agreed and believed in cCBT, and were confident about using it. One of the main reasons for this lack of knowledge is the lack of availability of cCBT programs in Arabic. Moreover, it appears that lack of knowledge about cCBT is not limited to Saudi Arabia, as this issue has also been demonstrated in other regions [7,24]. Nevertheless, this lack of knowledge does not greatly influence the attitude toward using computers, which is somewhat similar to the findings in Australia [25]. This suggests that lack of knowledge about cCBT is easy to overcome and does not affect the attitude toward cCBT. However, the authors of the Australian study concluded that it is possible to change the knowledge of mental health workers about cCBT, at least to some extent, by providing them with accurate information and demonstrations as needed [25]. In another study, the attitude toward computer usage showed a positive trend, which may provide more opportunities to benefit from cCBT [26]. These results indicate a promising future for more involvement with this approach [18,20,21].

In another study, Computer-Based Training Attitudes Scale scores were higher among therapists who reported having previously used computer-based training. Negative responses toward computer-based training largely originated from those facing greater practical barriers to the use of computer-based training [26]. In our study, there was no difference in cCBT knowledge between professionals with different backgrounds (eg, psychologists, psychiatrists, social workers, and others), which is similar to the results of the Australian study [25]. The reason could be that there is no difference in teaching and training among different specialties regarding cCBT. Most of our participants were unsure of their answers when asked about cCBT, which is similar to the results reported by Donovan et al [25] in Australia, as most of their participants had little to no knowledge of cCBT.

Clinically, cCBT can be beneficial. Reviewed advantages include flexibility in time and location, cost-effectiveness, reduction of personal stigma, time of the mental health care professional, time of waiting for treatment, the behavior of asking for help and guidance, and being satisfied by the provided treatment [30]. Despite the benefits mentioned, to accept cCBT as an alternative to the usual approach, more research should focus on how it works as an intervention [30]. There are some noted disadvantages of cCBT. Some primary concerns raised are the possible inadequacy of the therapeutic relationship and omission of the professional’s contact. Another concern is that the program is not customized to each user [21]. Moreover, some professionals doubt the competence of care provided through computers [30], and the majority still consider that the usual intervention is more effective and preferable [18,20,21].

### Future Directions

Future research should aim to recruit greater numbers of participants with various levels of training, skills, and different backgrounds so that effective comparisons can be made [25]. Future research should also provide clinicians with a chance to use cCBT with their clients to investigate if they would choose it. This could help generate a behavioral measure rather than assessing “intention” only [25]. The knowledge assessment in our study was very narrow, with only six questions. Future research should include a larger number of questions assessing knowledge to ensure that any information the participants may have is reflected in their knowledge scores [25]. In addition to the suggestions for future research, replicating the study several times over the next 10 years would be interesting as changes in the country are occurring rapidly [25]. In addition, assessing patients’ attitudes in Saudi Arabia toward cCBT would be very helpful.

### Limitations

We used an electronic questionnaire to reach the participants in Riyadh through data collectors. We did not receive a sufficient number of responses because of the limited mental health care professionals in Riyadh, and therefore we reached out to different cities in Saudi Arabia by sending an invitation through WhatsApp groups and emails. Future research should expand this approach by including a larger participant population. Future research should also provide participants with a broader range and more items in the knowledge test.

### Conclusions

The results of this study suggest that mental health care professionals in Saudi Arabia are in need of more education and training regarding cCBT; however, their attitudes toward its use, and their comfort in using computers in general, show great promise.

Lack of knowledge did not affect the participants’ attitude toward cCBT, as they demonstrated a positive attitude overall. In addition, we recognize that mental health care professionals need more involvement in various up-to-date therapeutic approaches and need more resources for cCBT in Arabic. Further research should be performed to assess patients’ acceptance of cCBT in Saudi Arabia along with clinical trials measuring its effectiveness in the Saudi population.

## Abbreviations

CBT: cognitive behavioral therapy
cCBT: computerized cognitive behavioral therapy
